# Genomic Characterization of a *Proteus* sp. Strain of Animal Origin Co-Carrying *bla*_NDM-1_ and *lnu(G)*

**DOI:** 10.3390/antibiotics10111411

**Published:** 2021-11-18

**Authors:** Ying Li, Yichuan Qiu, Junping She, Xu Wang, Xiaoyi Dai, Luhua Zhang

**Affiliations:** 1 School of Basic Medical Sciences, Southwest Medical University, Luzhou 646000, China; Lying1019@swmu.edu.cn (Y.L.); 20200199120016@stu.swmu.edu.cn (Y.Q.); shejunping@sina.com (J.S.); wangxu322@163.com (X.W.); 2 Immunological Technology Platform, Southwest Medical University, Luzhou 646000, China; 3 Pathogen Biology Platform, Southwest Medical University, Luzhou 646000, China

**Keywords:** carbapenem, IS*CR1*, pPvSC3-like, IS*Pst2*, Tn*6260*

## Abstract

The emergence of carbapenem-resistant *Proteus* represents a serious threat to global public health due to limited antibiotic treatment options. Here, we characterize a *Proteus* isolate NMG38-2 of swine origin that exhibits extensive drug resistance, including carbapenems. Whole-genome sequencing based on Illumina and MinION platforms showed that NMG38-2 contains 24 acquired antibiotic resistance genes and three plasmids, among which, pNDM_NMG38-2, a pPvSC3-like plasmid, is transferable and co-carries *bla*_NDM-1_ and *lnu(G)*. Sequence analysis of pPvSC3-like plasmids showed that they share a conserved backbone but have a diverse accessory module with complex chimera structures bearing abundant resistance genes, which are facilitated by transposons and/or homologous recombination. The acquisition of *bla*_NDM-1_ in pNDM_NMG38-2 was due to the IS*CR1*-mediated integration event. Comprehensive analysis of the *lnu(G)-*bearing cassettes carried by bacterial plasmids or chromosomes revealed a diversification of its genetic contexts, with Tn*6260* and IS*Pst2* elements being the leading contributors to the dissemination of *lnu(G)* in *Enterococcus* and Enterobacteriaceae, respectively. In conclusion, this study provides a better understanding of the genetic features of pPvSC3-like plasmids, which represent a novel plasmid group as a vehicle mediating the dissemination of *bla*_NDM-1_ among bacteria species. Moreover, our results highlight the central roles of Tn*6260* and IS*Pst2* in the spread of *lnu(G)*.

## 1. Introduction

*Proteus* is a member of Enterobacteriaceae and ranks third in regard to the cause of hospital-acquired infections [1]. *Proteus* spp. are important opportunistic pathogens that are commonly found in the environment and in the normal gut biota of humans and animals [1]. *Proteus* species are intrinsically resistant to polymyxin, tigecycline, and nitrofurantoin [2], and the acquisition of additional carbapenem resistance is of particular concern. In particular, strains producing the New Delhi metallo-β-lactamase (NDM), which is able to hydrolyze almost all of the β-lactams (including carbapenems), except monobactam, severely limit therapeutic options for treating infections caused by them [3]. The *bla*_NDM_ gene, since its initial identification in India in 2009 [4], has spread worldwide. The mobilization of *bla*_NDM_ is primarily associated with an IS*Aba125*-bounded composite transposon Tn*125* [3]. However, a variety of complex genetic contexts of *bla*_NDM_ have been identified, and mobile genetic elements, such as IS*26* [5], IS*903* [6], MITE [7], and IS*CR1* [8], were found to mobilize *bla*_NDM_. The *bla*_NDM_ gene is largely carried on plasmids with a variety of replicon types [3], which play a vital role in the wide dissemination of *bla*_NDM_. In *Proteus* spp., *bla*_NDM_ was either integrated into an SXT/R391 integrative and conjugative element [9,10] on bacterial chromosomes, or carried on plasmids with unknown replicon types, such as the recently reported pPrY2001-like plasmids [11,12]. In fact, the genetic characters of *bla*_NDM_-harboring plasmids in *Proteus* remain largely uncharacterized.

Lincosamide antibiotics, including lincomycin, clindamycin and pirlimycin, are potently active against Gram-positive cocci (*Staphylococcus*, *Streptococcus,* and *Enterococcus*) and anaerobic bacteria [13]. Lincomycin is commonly used in veterinary practice, alone or in combination with spectinomycin, and clindamycin is approved to treat infections in human medicine [14]. Lincosamides prevent protein synthesis by inhibiting the peptidyl transferase, by mainly binding to the key nucleotide A2058 of 23S rRNA in the 50S subunit of the bacterial ribosome [15]. Two major mechanisms are responsible for resistance to lincosamides: (i) methylation of 23S rRNA, a common mode of action for resistance to macrolides, lincosamides, and streptogramin B (MLS phenotype) [16]; (ii) enzymatic inactivation of lincosamides due to nucleotidylation by O-nucleotidyltransferase, conferring resistance to lincosamides only (L phenotype) [17]. Since the first report of lincosamide nucleotidyltransferase encoded by *lnu(A)* (formerly *linA*) [17], several *lnu* genes, including *lnu(B)*, *lnu(C)*, *lnu(D)*, *lnu(E)*, *lnu(F)*, *lnu(G)*, and *lnu(P)*, were subsequently identified in animal and human isolates [14,18,19,20,21]. Among them, *lnu(G)* was first identified in a swine *Enterococcus faecalis* isolate WT E531 in 2016 in China, and it was embedded in an active Tn*6260* [14]. In silico analysis using the GenBank database indicated that *lnu(G)* was presented in a number of isolates recovered worldwide. To reveal the dissemination patterns of *lnu(G)*, a systematic analysis of genetic contexts of *lnu(G)* in different plasmids and chromosomes is required.

The present study was conducted to characterize a carbapenem-resistant *Proteus* sp. isolate of animal origin. This strain harbors a *bla*_NDM-1_ gene that is located on a pPvSC3-like plasmid with an unknown replicon type [22]. We elucidate the complete sequence of this *bla*_NDM-1_-bearing plasmid and compare it with two other similar plasmids to obtain insights into the mechanisms mediating the mobilization of *bla*_NDM-1_ and the diversification of these plasmids. In addition, we analyze the genetic features of *lnu(G)* systematically to gain a deeper understanding of the complex and diversified dissemination patterns of this important resistance gene.

## 2. Results and Discussion

### 2.1. Genetic Characterization of the bla_NDM-1_-Harboring Proteus sp. NMG38-2

A carbapenem-resistant strain, named NMG38-2, was recovered from one of the 40 fecal samples of swine. Whole-genome analysis revealed that NMG38-2 contains a single circular chromosome of 4,433,346 bp, with an average GC content of 37.62%. It also carries three circular plasmids, designated as pNDM_NMG38-2, p1_NMG38-2, and p2_NMG38-2. The species identification was performed by genome-to-genome sequence comparison of NMG38-2 and the reference strains of known *Proteus* species. As a result, the average nucleotide identity (ANI) values between NMG38-2 and known *Proteus* species were all ≤91.23%, which is below the recommended 95% cut-off for species circumscription. Consistent with ANI data, the digital DNA-DNA hybridization (dDDH) identities between the genome of NMG38-2 and those of reference *Proteus* species were ≤44.7%, below the suggested 70% cut-off to define a species. Both ANI and dDDH values indicated that the NMG38-2 belongs to a novel species that is distinct from all hitherto known *Proteus* species. ANI and dDDH analysis also revealed that NMG38-2 showed a higher identity (ANI value: 91.23%, dDDH value: 44.7%) with the *Proteus vulgaris* strain ATCC 49132 (GenBank accession no. NZ_KN150745.1) than with the other known *Proteus* species.

While being intrinsically resistant to polymyxin and tigecycline, *Proteus* sp. NMG38-2 was extensively drug resistant [23], exhibiting resistance to all tested antimicrobials except ciprofloxacin (Table 1). Consistent with its multidrug resistance profile, NMG38-2 contained 24 intact acquired antibiotic resistance genes (ARGs), conferring resistance to aminoglycosides (*ant(2″)-Ia*, *aph(6)-Id*, *aac(6′)-Ib3*, *aadA1*, *aph(3″)-Ib,* and *aac(6′)-Ib-cr*), rifampicin (*ARR-3*), sulfonamides (*sul1* and *sul2*), trimethoprim (*dfrA1*), quinolones (*aac(6′)-Ib-cr* and *qnrD1*), phenicol (*catB8, catB3, catB2,* and *floR*), β-lactams (*bla*_OXA-10_ and *bla*_NDM-1_), macrolide (*mph(E)* and *msr(E)*), and lincosamide (*lnu(G)*) (Table 2). These ARGs were mainly located on the plasmid pNDM_NMG38-2, except that the chromosome harbored an *aadA1*, *dfrA1,* and *catB2*, and the *qnrD1* was carried by the small plasmid p2_NMG38-2. We proposed that the extensive use of drugs might contribute to the emergence of the extensively drug resistant strain, for the reason that multiple antibiotics, including amoxicillin, ceftiofur, sulfamethazine sodium, gentamycin sulfate, and so on, were used within one month prior to sample collection. The abusive use of antimicrobials in animal husbandry, swine industry in this case, is believed to be an important driving force for the emergence and transmission of ARGs in bacteria of animal origin [24], which may further act as the potential reservoir of ARGs for clinical pathogens resistant to multiple antibiotics, including carbapenems.

The *bla*_NDM-1_-bearing plasmid pNDM_NMG38-2 is 262,556 bp in size with an average G+C content of 46.65%, and encodes 369 open reading frames (ORFs, Table 2). This plasmid could not be assigned to any known incompatibility group. BLAST analysis of pNDM_NMG38-2 against the GenBank database revealed that it showed the highest 98% query coverage and 99.99% identity to pM2-1 (GenBank accession no. MT813046), a *bla*_NDM-1_-harboring plasmid in a *Providencia stuartii* strain isolated from mouse feces from a commercial goose farm in China [22] (Table S1). It also showed 99.98% identity with 92% query coverage to a *bla*_NDM-1_-negative plasmid pPvSC3 (accession no. CP034667) in a *P. vulgaris* isolate from a chicken in China [25] (Table S1). No other similar plasmid was found in the GenBank database. Using the nucleotide sequence of the replication gene *repA* of pNDM_NMG38-2 as a query, matches with 100% query coverage and at least 99% identity were pM2-1 and pPvSC3. Thus, the three plasmids, pM2-1, pPvSC3, and pNDM_NMG38-2 were assigned into the same unknown incompatibility group. pPvSC3 is the earliest reported plasmid and considered to be the reference plasmid, so these three plasmids are called pPvSC3-like plasmids. Notably, the pPvSC3-like plasmids were all recovered from isolates of animal origins from different regions of China, highlighting that this novel plasmid group may serve as an epidemic vehicle in mediating the dissemination of *bla*_NDM-1_ in animals in China. Continuous surveillance on the spread of pPvSC3-like plasmids in humans and the environment in China is urgently required.

p1_NMG38-2 is a 5500 bp plasmid with a mean G+C content of 34.11% and contains 10 predicted ORFs (Table 2). It did not belong to any known incompatibility group. BLAST analysis revealed that p1_NMG38-2 was partial homology to four plasmids, pZF2-7kb (CP047342, *Proteus terrae*, swine, China), pZF2-7kb (CP045010, *P. terrae*, swine, China), pLC-693_2 (CP063316, *P. vulgaris*, patient, Switzerland), and pOXA181-15-1091(CP012904, *Providencia rettgeri*, patient, Canada), with only 49–67% coverage and 90.67–92.53% nucleotide identity, indicating that p1_NMG38-2 was a novel plasmid. This plasmid encodes a replication protein RepB belonging to the Rep_3 type family, a DNA-binding protein and a putative toxin-antitoxin system RelE/RelB for plasmid maintenance, as well as six hypothetical proteins. p2_NMG38-2 is 2683 bp in length with a 41.78 GC content (Table 2). It harbors two predicted ORFs, including a resistance gene *qnrD1,* and belongs to the Col3M incompatibility group. p2_ NMG38-2 shared high similarity (100% coverage, >99.9% identity) to a number of plasmids in the GenBank database, such as pMP63B in *Morganella morganii* (CP048808, wastewater, China), plasmid unnamed2 in *Proteus mirabilis* (CP049755, patient, Brazil), pRHB14-C12_2 in *Citrobacter freundii* (CP057825, swine, United Kingdom), and pAB213 in *P. rettgeri* (MH085193, vegetable, Brazil), suggesting a wide spread of p2_NMG38-2-like plasmids in various niches worldwide.

### 2.2. Phylogenetic Analysis of Proteus sp. NMG38-2

**Figure 1:**
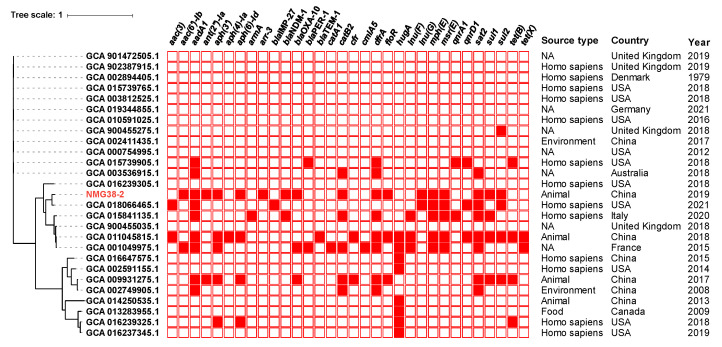
Phylogenetic tree of the *Proteus* sp. NMG38-2 with 26 *P. vulgaris* genomes available from GenBank. The tree is based on 1323 core genes with 232,421 SNPs and the tree scale indicates substitutions per site. NMG38-2 is indicated in red. Resistance gene profiles are visualized in compliance to the tree. The annotation denotes (from left to right) isolation sources, locations and years of strains. NA, not available.

To determine a possible clinical relevance of NMG38-2 and explore the evolutionary relationships between NMG38-2 and *P. vulgaris* strains, phylogenetic analysis based on core genome alignment of NMG38-2 and all 26 available *P. vulgaris* strains from GenBank (on 2 September 2021), was performed. In total, 1323 core genes with 232,421 single nucleotide polymorphisms (SNPs) were identified from these genome sequences. These *P. vulgaris* isolates were mainly recovered from clinical specimens around the world, and found in the animals and environment. The phylogenetic tree (Figure 1) depicted a partitioning into three groups, and NMG38-2 was clustered with isolates of human and animal origins across various countries (including USA, Italy, France, United Kingdom, and China). NMG38-2 had the most closely genetic relationship with a clinical strain (GenBank assembly accession number GCA_018066465.1) from a patient with leg wound in USA in 2021, with 15,188 SNPs difference. This result indicated a close clinical relevance of NMG38-2. Resistance genes profiles showed that NMG38-2 and other strains in the same branch carried significantly more ARGs than the other two groups. A sporadic acquisition of carbapenemase genes was identified in these *Proteus* strains, with NMG38-2 and a clinical isolate from Italy in 2020 representing the only two strains harboring *bla*_NDM-1_.

### 2.3. Genetic Structure of pPvSC3-like Plasmids

**Figure 2:**
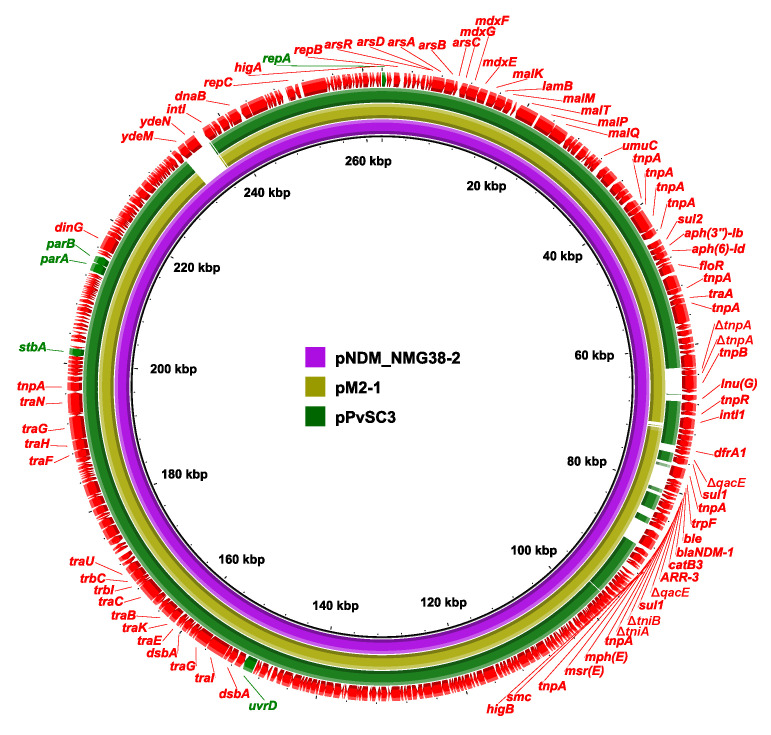
Circular comparisons of pNDM_NMG38-2 with pPvSC3 and pM2-1. pNDM_NMG38-2 was used as a reference to compare with other plasmids. The outer circle with red arrows denotes the annotation of reference plasmid, with arrows indicating deduced ORFs and their orientations. Gaps indicate regions that were missing in the respective plasmid compared to the reference plasmid. Key backbone genes are indicated in green.

**Figure 3:**
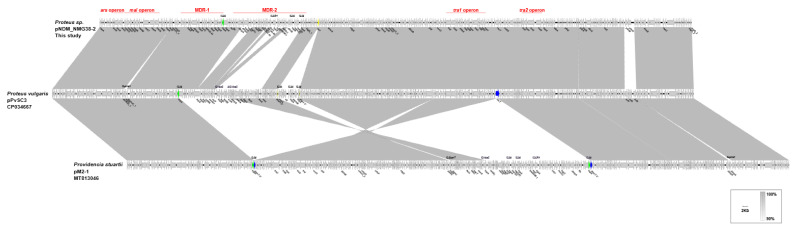
Linear comparisons of pNDM_NMG38-2, pPvSC3, and pM2-1. The *tra1*, *tra2* operon, *mal* operon, *ars* operon, MDR-1, and MDR-2 regions are pointed out. Arrows indicate deduced ORFs and their orientations. Gray shading denotes regions of shared homology ranging from 90% to 100%. Δ represents truncated genes or mobile genetic elements.

Pairwise sequence comparison using BLASTN showed that these three plasmids had almost identical backbone (>99.98% nucleotide identity across >96% sequences, nucleotide (nt) 1 to 37,670 bp and 97,255 to 262,556 bp of pNDM_NMG38-2, Table S2, Figure 2 and Figure 3). The major backbone genes included *repA* and its iterons for replication, *parAB* and *stbA* for plasmid partition, *umuCD* and *uvrD* for plasmid maintenance, *tra1* and *tra2* operon for conjugal transfer (Figure 2 and Figure 3). These three plasmids also harbored the *mal* operon for maltose metabolism and *ars* operon for arsenic resistance in the backbone region (Figure 2 and Figure 3). There were minor differences among their backbones: (i) the *umuC_1* gene in pPvSC3 and pM2-1 is disrupted into Δ*umuC_1-5′* and Δ*umuC_1-3′* by an insertion of IS*prre1*; (ii) the *orf1-orf3-orf6* segment (nt 84,819–89,762 bp) in pPvSC3 was replaced by an *ydeM*-*ydeN* structure (encoding sulfatase and sulfatase-maturating enzyme) in pNDM_NMG38-2; and (iii) an IS*prre1* was inserted at a site downstream of the *orf1-orf3-orf6* segment in pM2-1.

**Figure 4:**
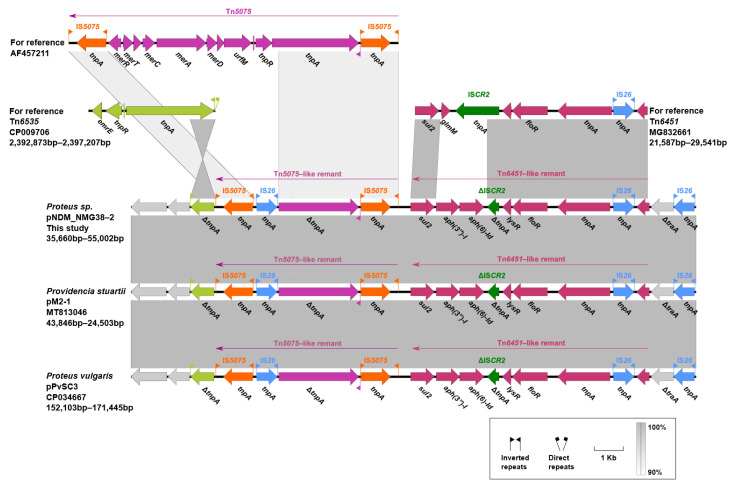
Organization of the MDR-1 of pPvSC3-like plasmids and comparison to related regions. Genes are denoted by arrows. Genes, mobile elements, and other features are colored based on their functional classification. Gray shading denotes regions of shared homology ranging from 90% to 100%. Δ represents truncated genes or mobile genetic elements.

**Figure 5:**
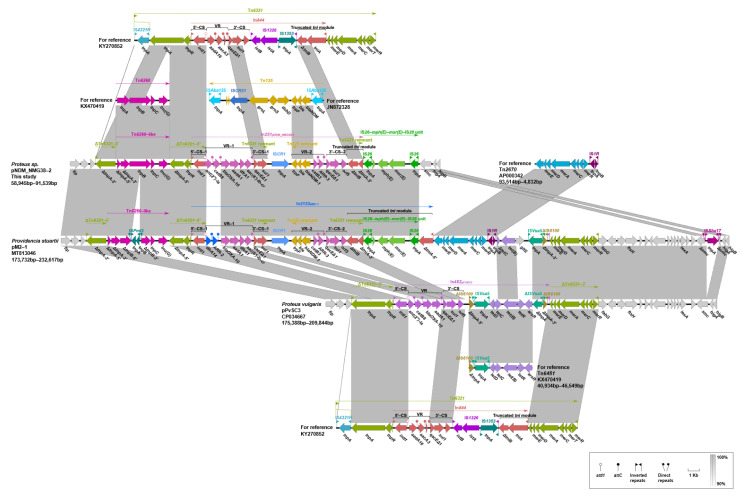
Organization of the MDR-2 of pPvSC3-like plasmids and comparison to related regions. Genes are denoted by arrows. Genes, mobile elements, and other features are colored based on their functional classification. Gray shading denotes regions of shared homology ranging from 90% to 100%. Δ represents truncated genes or mobile genetic elements.

Two resistance accessory modules, designated MDR-1 and MDR-2, were identified in pPvSC3-like plasmids. The MDR-1 region was highly similar in these three plasmids with 100% nucleotide identity across >98.97% sequences. It had a chimera structure comprising of segments of Tn*6535*, Tn*5075,* and Tn*6451* (Figure 4). This region contained four ARGs, including *aph(3″)-Ib*, *aph(6)-Id*, *sul2,* and *floR*, and an abundance of mobile genetic elements, which were responsible for the formation of this mosaic MDR region. The MDR-2 regions found in these three plasmids were identified as derivatives of Tn*6321*, which was a unit transposon belonging to the Tn*21* subgroup of Tn*3* family, and was generated by an insertion of a class 1 integron In*844* at a site downstream of the *tnpA-tnpR*-*res* module in the primary backbone structure (Figure 5). Various types of insertion or homologous recombination events occurred within these MDR-2 regions, leading to gene truncations as well as the integration of foreign resistance markers.

Differences in three major modulars in the MDR-2 region, namely the *tnpA-tnpR-res,* integrons and *mer*, were identified (Figure 5). First, a core *lnu(G)* platform, Tn*6260-*like structure, was inserted into the transposase gene of Tn*6321*, truncating and breaking *tnpA* into separate Δ*tnpA-5′* and Δ*tnpA-3′* in both pNDM_NMG38-2 and pM2-1. The Tn*6260-*like structure was different from Tn*6260* by an internal termination of *tnpA* gene caused by a base mutation (C to T at the 280th base). The *tnpB* gene of Tn*6260* was further disrupted by an insertion of IS*Pmi3* in pM2-1. Second, the integron-related region showed distinct profiles: (i) compared to the In*462* in pPvSC3, a “complex” class 1 integron harboring *bla*_NDM-1_ was found in pM2-1 and pNDM_NMG38-2. (ii) A novel integron, designated In*2123*, was identified in pM2-1, which consists of one 5′-conserved segment (5′-CS: *intI1-attI1*), two 3′-conserved segments (3′-CSs: *qacED1*-*sul1*-*orf5*-*orf6)*, one IS*CR1*, two variable regions (VRs, *dfrA14-ARR-2-bla*_OXA-10_-*aadA1*-*dfrA1*-*aac(6′)-Ib-cr* in VR1 and *trpF-ble-bla*_NDM-1_-*catB3*-*ARR-3* in VR2), an IS*26-mph(E)*-*msr(E)*-IS*26* unit and a *tni* region truncated by the IS*26* unit. (iii) In pNDM_NMG38-2, an In*251* derived “complex” class 1 integron was found, which was similar to the In*2123* in pM2-1, except that the gene cassettes *dfrA14*-*ARR-2* was replaced by *ant(2″)-Ia*-*catB8*, and the *tniA*-*5′* as well as the IRt (IR at the *tni* end) was deleted in pNDM_NMG38-2. Third, the *mer* region varies greatly in three plasmids: (i) a Tn*6451* remnant containing *tet* gene clusters (*tetDCBR*) related to tetracycline resistance was inserted at a site within the IS*6100* upstream of the *mer* region mediated by IS*Vsa5* in pPvSC3. (ii) A Tn*2670* remnant comprising of *mer* gene clusters (*merEDACPTR*) followed by a mobile element IS*1R* and a fragment containing an Δ*tetC-tetB-gltS-*IS*Vsa5-*ΔIS*6100* module were successively integrated upstream of the *mer* region of Tn*6321* in pM2-1, with two *mer* gene clusters showing 99% nucleotide coverage and 81% identity. (iii) Compared to pM2-1, a 24,455-bp region covering the *tniA*-*5′*, the complete *mer* region, as well as the surrounding backbone region was deleted in pNDM_NMG38-2, which was most likely due to the IS*26-*mediated recombination.

It is notable that an IS*26*-bounded structure (IS*26*-*cfr*-IS*26*-*ΔermB*-IS*26*), bearing the multiresistance gene *cfr*, was inserted within a hypothetic gene *hp1* at 15,730 bp downstream of the *mer* region, truncating and splitting it into two parts *Δhp1-5′* and *Δhp1-3′* in pPvSC3 (Figure 3). Additionally, an IS*26* was inserted at a site within the *recT* gene in pM2-1, breaking it into two segments *ΔrecT-5′* and *ΔrecT-3′.* The inserted IS*26* within *recT*, and the one at the right-hand end of MDR-1 region, were most likely to have accounted for the subsequent inversion of the 144,246-bp fragment covering MDR-2 and its surrounding backbone regions, by complex homologous recombination events (Figure 3). In conclusion, pPvSC3-like plasmids share a conserved backbone, but possess highly genetic plasticity in the MDR-2 region, which harbors a large number of resistance genes that are associated with insertion sequences, integrons, and transposons. The IS*CR* element, which is known to mobilize associated resistance genes via a rolling-circle mechanism [26], plays a key role in the acquisition of *bla*_NDM-1_ in pNDM_NMG38-2 and pM2-1.

### 2.4. Transfer Ability of pNDM_NMG38-2 and Fitness Cost

Conjugation assays showed that pNDM_NMG38-2 was able to conjugate into *E. coli* J53 at a frequency of ~10^−6^. Antimicrobial susceptibility results showed that the acquisition of pNDM_NMG38-2 greatly increased meropenem and imipenem resistance in J53 by at least 1024-fold. These results indicate that plasmid pNDM_NMG38-2 was self-transmissible, confirming that pPvSC3-like plasmids are transferable [22,25]. Growth curves showed that no significant differences in the growth rates were observed in both lag and logarithmic phases among the recipient strain J53 and transconjugants (Figure S1), suggesting that the carriage of pNDM_NMG38-2 does not significantly exert the fitness cost in its recipient host.

### 2.5. Genetic Contexts of lnu(G)

A total of 53 *lnu(G)*-bearing plasmids and chromosomes were retrieved from the GenBank database (accessed on 18 August 2021, Table S3). *lnu(G)* has a wide host spectrum of different origins with a worldwide distribution. It is largely located on the chromosomes of *Enterococcus* spp., and sporadically found on those of *Providencia* sp., *M. morganii*, *P. mirabilis* etc. Besides, *lnu(G)* is carried on plasmids that are widespread in Enterobacteriaceae, with *Escherichia coli* being the most common species, followed by *Proteus* spp. It is also identified on plasmids in bacterial species including *Acinetobacter variabilis*, *Listeria monocytogenes*, *P. rettgeri*, and *E. faecalis*. Plasmids with a variety of replicon types are associated with the dissemination of *lnu(G)* (Table S3). Among them, hybrid plasmids containing replicons of IncFIA(HI1), IncHI1A, and IncHI1B(R27), or in combination with other types of replicons, such as IncFII or IncX4, are most commonly found to carry *lnu(G)* in Enterobacteriaceae.

**Figure 6:**
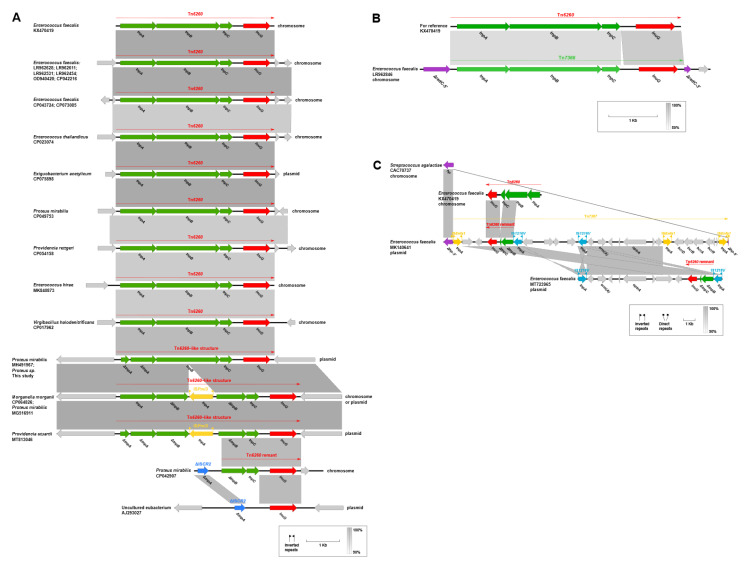
Tn*6260*-associated genetic contexts of *lnu(G)* from different plasmids and chromosomes. The *lnu(G)* was indicated in red. Genes are colored based on their functional classification. Gray shading denotes genetic regions that exhibit sequence homology among different segments. Δ represents truncated genes or mobile genetic elements.

**Figure 7:**
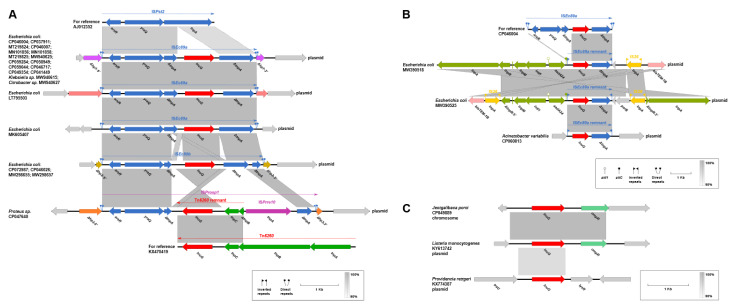
The genetic contexts of *lnu(G)* from other plasmids and chromosomes. IS*Pst2*-associated genetic contexts of *lnu(G)*, type D1 and type D2, are shown in (**A**) and (**B**), respectively. The *lnu(G)* genetic contexts of type E is shown in (**C**). The *lnu(G)* was indicated in red. Genes are colored based on their functional classification. Gray shading denotes genetic regions that exhibit sequence homology among different segments. Δ represents truncated genes or mobile genetic elements.

The genetic features of *lnu(G)* showed diversification and could be categorized into five different types, designated A (*n* = 22), B (*n* = 1), C (*n* = 2), D (*n* = 26), and E (*n* = 3) (Figure 6A–C and Figure 7A–C).

**Type A:** Tn*6260*, first identified on the chromosome of a swine *E. faecalis* (accession no. KX470419) [14], is a major vehicle in mediating the dissemination of *lnu(G).* An intact Tn*6260* was particularly common in *Enterococcus* and was identified in other bacterial species (Figure 6A).

Tn*6260-*like structures found in *P. mirabilis* strains (accession no. MH491967 and MG516911), *Proteus* sp. NMG38-2, *M. morganii* (accession no. CP064826), and *P. stuartii* (accession no. MT813046) were different from the wild type transposon by an internal termination of *tnpA* gene or/and an interruption of *tnpB* gene by the insertion of IS*Pmi3* (Figure 6A).

In a *P. mirabilis* (accession no. CP042907), the complete *tnpA* and partial *tnpB* (*tnpB-5′*) of Tn*6260* was lost, and a truncated IS*CR2* took their places. Genetic events by IS*CR2* might have contributed to the disruption of Tn*6260.* On this basis, the *tnpB* remnant (*tnpB-3′*) together with the downstream *tnpC* was further deleted in a plasmid from an uncultured Eubacterium (accession no. AJ293027) (Figure 6A).

**Type B:** on the chromosome of an *E. faecalis* (accession no. LR962846), a novel *lnu(G)*-bearing transposon, designated Tn*7366*, was inserted into the *radC* gene, splitting it into two separate parts (Figure 6B). It exhibits 98% nucleotide coverage and 90.37% identity to Tn*6260*, with 92.36% (100% coverage), 91.16% (99% coverage), and 81.79% (100% coverage) identities to *tnpA*, *tnpB*, and *tnpC*, respectively.

**Type C:** a novel *lnu(G)*-bearing composite transposon bounded by two copies of IS*Enfa1*, designated Tn*7367*, was detected in a plasmid from an *E. faecalis* isolate (accession no. MK140641), where it was inserted into a hypothetical gene and leaved a paired terminal 6-bp direct repeats (DRs, target site duplication signals for transposition, TTTTTA, Figure 6C). In Tn*7367*, a Tn*6260* remnant containing a 1011-bp *tnpB* (*tnpB-3′*), a 123-bp *tnpC* (*tnpC-5′*) and the intact *lnu(G)* was integrated into a site downstream of IS*1216V* near the IS*Enfa1* at the left-hand. An identical Tn*6260* remnant was also found to be carried by a plasmid from another *E. faecalis* strain (accession no. MT723965); however, in this isolate, it was located in a region bracketed by two IS*1216V* in the opposite orientation (Figure 6C). This IS*1216V* bounded region was most likely to assemble from a structure like Tn*7367* by multiple homologous recombination events of IS*1216V*.

**Type D1:** another common mechanism mediating the wide spread of *lnu(G)* was associated with IS*Pst2*, a member of the ISL3 family, which was widely identified in *Pseudomonas stutzeri* strain OX1 [27]. The IS*Pst2* is composed of three genes, *tnpA*, *yraQ,* and *arsR*, encoding a transposase, putative permease, and an ArsR family transcriptional regulator, respectively. *lnu(G),* together with its flanking sequences, was integrated into a site within the *tnpA* gene by an unknown genetic event (Figure 7A). The resulting novel *lnu(G)-*bearing IS*L3*-like transposon, designated IS*Ec89a* or IS*Ec89b*, was primarily identified in Enterobacteriaceae strains, where it was inserted into various sites of plasmids, with the presence of paired terminal 8-bp DRs. The different integrating position of *lnu(G)* within *tnpA* represents the main modular difference between IS*Ec89a* and IS*Ec89b*. Another *lnu(G)-*bearing transferable element, designated IS*Prosp1*, was detected in a plasmid from a *Proteus* sp. (accession no. CP047640), where it splits a hypothetic gene into two separate parts and meanwhile leaves 8-bp DR (TTTTTTTG). IS*Prosp1* was generated from integration of an IS*prre10* of the IS*91* family and its linked Tn*6260* remnant covering a 144-bp *tnpB* residue (*tnpB-3′*), the intact *tnpC* and *lnu(G)* into the *tnpA* gene of IS*Pst2.*

**Type D2:** an IS*Ec89a* remnant covering the inverted repeat left (IRL), the intact *lnu(G)*, a 924-bp *tnpA* fragment (*tnpA-3′*), and inverted repeat right (IRR) was found to be integrated into the cassette region, downstream of *aadA24*, of a class 1 integron carried by plasmids from two *E. coli* strains (accession no. MW390518 and MW390525, (Figure 7B). The identical IS*Ec89a* remnant was also detected in a plasmid from an *Acinetobacter variabilis* strain (accession no. CP060813), where it was inserted at a site between two genes encoding for a putative integrase and a recombinase, respectively. The detection of the same IS*Ec89a* remnant in different plasmids from different bacterial species indicates a possible transposition event of this *lnu(G)-*bearing element.

**Type E:** both on a plasmid from a *L. monocytogenes* strain (accession no. KY613742) and the chromosome of a *Jeotgalibaca porci* strain (accession no. CP049889), *lnu(G)* was linked to an *ompR* gene (encoding an ABC transporter-like sensor linked response regulator), whereas there was no mobile genetic element found adjacent to the *lnu(G)-ompR* structure (Figure 7C). Similarly, a *lnu(G)* was detected in a plasmid from a *P. rettgeri* isolate (accession no. KX774387), where it was located between *traU* (encoding a conjugal transfer pilus assembly protein) and *lysR* (encoding a transcriptional regulator) in the plasmid backbone region. The mechanisms involved in the mobilization of *lnu(G)* in these cases remain unknown.

Taken together, Tn*6260* serves as a major vehicle in mediating the dissemination of *lnu(G)* in *Enterococcus*, while IS*Pst2* element is the leading contributor to the mobilization of *lnu(G)* in Gram-negative bacteria, especially Enterobacteriaceae.

## 3. Materials and Methods

### 3.1. Bacterial Strain and In Vitro Susceptibility Testing

*Proteus* sp. NMG38-2 was isolated from an intensive pig farm in Inner Mongolia, China, in December 2018. This strain was recovered from a fecal sample of swine according to the method described previously, with minor modifications [28]. A total of 40 non-duplicate fecal samples from healthy pigs were collected and feces suspension was directly plated on MacConkey agar plates containing 2 μg/mL meropenem using sterile swabs. Colonies recovered were further purified in LB agar plates and broth (Sangon Biotech, Shanghai, China) containing 2 μg/mL meropenem and initial species identification was performed by PCR amplifying of 16S rRNA gene and Sanger sequencing [29]. The minimum inhibitory concentrations (MICs) of the following antimicrobials against *Proteus* sp. NMG38-2 were determined using the microdilution broth method, according to the Clinical and Laboratory Standards Institute (CLSI) guidelines [30]: amikacin, gentamicin, meropenem, imipenem, cefoxitin, aztreonam, ciprofloxacin, cefotaxime, clindamycin, colistin, and chloramphenicol. *E. coli* ATCC 25922 served as a quality control for MIC determination. All antibiotics used in this study were obtained from Yuanye Bio-Technology Co. (Shanghai, China).

### 3.2. Conjugation Assay

Conjugation experiments were performed using broth-based methods as described previously with minor modification [31]. The azide-resistant *E. coli* strain J53 was used as the recipient. Both the NMG38-2 and J53 were grown to exponential stage (the optical density at 600 nm reaches ~0.5) and then mixed at a donor/recipient ratio of 1:1. After incubation at 37 °C for 24 h, transconjugants were selected on LB agar plates containing 2 μg/mL meropenem plus 150 μg/mL sodium azide. The presence of *bla*_NDM-1_ in transconjugants was confirmed by PCR using the primers *bla*_NDM_-F 5′-ATTTACTAGGCCTCGCATTTGC-3′ and *bla*_NDM_-R 5′-GCCTCTGTCACATCGAAATCG-3′.

### 3.3. In Vitro Growth Assays

Three independent cultures of J53 and transconjugants harboring pNDM_NMG38-2 were grown overnight and diluted to 1:100 in LB broth. Bacteria cultures were incubated while shaking (200 rpm) at 37 °C. In a total period of 12 h, the value of optical density (OD) at 600 nm (OD600) was consistently recorded at an interval of 1 h using the iMark microplate Reader (Bio-Rad, Hercules, CA, USA).

### 3.4. Genome Sequencing and Data Analyses

Genomic DNA of *Proteus* sp. NMG38-2 was extracted using the QIAamp DNA Mini Kit (Qiagen) and purified DNA was subjected to whole genome sequencing using the long-read MinION Sequencer (Nanopore, Oxford, UK), as well as a paired-end library with an insert size of 150 bp on a HiSeq 2000 sequencer (Illumina, San Diego, CA, USA). The de novo hybrid assembly of both long MinION reads and short Illumina reads was performed using Unicycler under the conservative mode [32]. Pilon was employed to correct the assembled contigs with Illumina reads for several rounds until no change was detected [33]. Annotations of the genome and plasmid sequences were conducted using the RAST tools [34] combined with BLASTp/BLASTn searches against the UniProtKB/Swiss-Prot database [35]. The ANI value between genome sequences of NMG38-2 and those of the reference strain of the known *Proteus* species was calculated with JSpeciesWS (http://jspecies.ribohost.com/jspeciesws/#analyse, accessed on 20 October 2021). dDDH values were calculated using GGDC 3.0 server (http://ggdc.dsmz.de/distcalc2.php, accessed on 20 October 2021) by means of genome-to-genome sequence comparison. The annotation of resistance genes, mobile elements, and other features was carried out using the online databases ResFinder [36], ISfinder [37], and INTEGRALL [38]. Alignments with homologous plasmid sequences of pNDM_NMG38-2 available in NCBI were performed by using the BRIG tool [39] and Easyfig v 2.2.3 (University of Queensland, Brisbane QLD 4072, Australia) [40] (accessed on 20 September 2021 to 7 October 2021). Gene organization diagrams were visualized with Inkscape 0.92.4 (https://inkscape.org/en/, accessed on 7 October 2021 to 17 November 2021).

### 3.5. Phylogenetic Analysis

Genomes were annotated using Prokka and annotated GFF3 files were piped into Roary to create a core genome alignment. SNPs were extracted using SNP-sites v2.3.2 (accessed on 12 September 2021) [41]. A maximum-likelihood phylogenetic tree based on the SNPs was constructed using FastTree version 2.1.10 (accessed on 12 September 2021) [42]. The antimicrobial resistance gene profiles were determined using ABRicate (https://github.com/tseemann/abricate, accessed on 16 September 2021). Carriage of resistance genes and detail information (source type, country, and isolation year) of isolates were annotated on the trees using iTOL (accessed on 17 September 2021) [43].

### 3.6. Comparative Analysis of lnu(G)-Harboring Genome Sequences

To obtain *lnu(G)*-harboring genome sequences, a nucleotide BLAST with standard options was performed in the NCBI GenBank database with the nucleotide base sequence of *lnu(G)* as a query. Models and uncultured environmental samples were excluded. Complete plasmids and chromosomes containing at least one contig with a full-length hit to *lnu(G),* with ≥99% query coverage and at least 97% identity, were selected and exported. Sequence alignments among *lnu(G)*-carrying plasmids or chromosomes were performed with Easyfig v 2.2.3 (accessed on 9 October 2021 to 14 October 2021) [40].

### 3.7. Nucleotide Sequence Accession Numbers

The complete genome sequences of *Proteus* sp. NMG38-2 and its plasmids have been deposited in GenBank under BioProject no. PRJNA769966.

## 4. Conclusions

In the present study, we characterized a drug resistant *Proteus* sp. from swine, which harbored *bla*_NDM-1_ carried on a conjugative plasmid of the untypable pPvSC3-like plasmid group. Our data significantly extended our understanding of genetic features of the pPvSC3-like plasmids, which could be a possible contributor to the spread of *bla*_NDM-1_ between *Proteus* and other species, in addition to the previously reported pPrY2001-like plasmids. More whole-genome sequencing-based epidemiological studies are warranted to achieve better insights into the mechanisms of the spread of carbapenemase genes in *Proteus.* Moreover, this work also allowed us to obtain a clear picture of the genetic contexts of *lnu(G)*, highlighting the central roles of Tn*6260* and IS*Pst2* in the spread of *lnu(G)* among diverse bacterial species.

## Figures and Tables

**Table 1:** MICs for NMG38-2, its transformants, and the recipient strain J53.

Strains ^a^	Species’ Name	MIC (μg/mL) ^b^										
		AMK	GEN	CST	MEM	IMP	CFT	AZT	CIP	CTX	CLN	CHL
NMG38-2	*Proteus* sp.	**128**	**256**	**512**	**512**	**>512**	**512**	**256**	≤0.5	**256**	**>512**	**256**
JM-1	*E. coli*	**64**	**32**	**256**	**512**	**>512**	**512**	**256**	1	**256**	**>512**	**256**
JM-2	*E. coli*	**64**	**32**	**512**	**512**	**>512**	**512**	**256**	1	**512**	**512**	**256**
JM-3	*E. coli*	**64**	**64**	**256**	**512**	**>512**	**512**	**128**	2	**256**	**512**	**256**
J53	*E. coli*	16	2	≤0.5	≤0.5	≤0.5	16	8	≤0.5	≤0.5	**256**	8
ATCC25922	*E. coli*	4	4	≤0.5	≤0.5	≤0.5	8	2	≤0.5	≤0.5	**256**	16

^a^ JM-1, JM-2 and JM-3 are transconjugants harboring pNDM_NMG38-2. ^b^ AMK, amikacin; GEN, gentamicin; CST, colistin; MEM, meropenem; IMP, imipenem; CFT, cefoxitin; AZT, aztreonam; CIP, ciprofloxacin; CTX, cefotaxime; CLN: clindamycin; CHL, chloramphenicol. Resistant MICs are highlighted in bold.

**Table 2:** Summary of the genetic features of *Proteus* sp. NMG38-2.

Chromosome /Plasmid	Length (bp)	GC%	No. of Predicted Open Reading Frames	Inc Type	Drug Resistance Gene
Chromosome	4,433,346	37.62	4231	-	*aadA1, dfrA1, catB2*
pNDM_NMG38-2	262,556	46.65	369	UT	*aac(6′)-Ib-cr, ant(2* *″* *)-Ia, aph(6)-Id, aac(6′)-Ib3, aph(3* *″* *)-Ib, aac(6′)-Ib-cr, aadA1, ARR-3, sul1 ^a^, dfrA1, sul2, catB8, floR, catB3, bla_OXA-10_, bla_NDM-1_, mph(E), msr(E), lnu(G)*
P1_SCLZS62	5500	34.11	10	UT	
P2_SCLZS62	2683	41.78	2	Col3M	*qnrD1*

^a^ Multiple copies on the plasmid. -, not available; UT, unknown type.

## Data Availability

The complete genome sequences of *Proteus* sp. NMG38-2 and its plasmids can be found in public NCBI Genbank databases under BioProject no. PRJNA769966.

## Supplementary Materials

The following are available online at https://www.mdpi.com/article/10.3390/antibiotics10111411/s1, Table S1: Pairwise comparison of complete sequences of pPvSC3-like plasmids using BLASTN. Table S2: Pairwise comparison of plasmid backbone sequences of pPvSC3-like plasmids using BLASTN. Table S3: Detailed information of *lnu(G)*-carrying plasmids or chromosomes (last accessed 18 August 2021). Figure S1: Growth curves for the transconjugants (JM-1, JM-2, and JM-3) and the recipient strain J53.
