# Supplementary material for: Genomic Characterization of a Proteus sp. Strain of Animal Origin Co-Carrying blaNDM-1 and lnu(G)
Source: Antibiotics (Basel). 2021 Nov 18;10(11):1411. doi: 10.3390/antibiotics10111411 (PMC8615141; doi:10.3390/antibiotics10111411)
Supplement: Supplementary file 1 [file antibiotics-10-01411-s001.zip › antibiotics-1441273-supplementary.pdf]

Table S1. Pairwise comparison of complete sequences of pPvSC3-like plasmids using BLASTN.

(coverage+identity)	pNDM_NMG38-2	pPvSC3	pM2-1
**pNDM_NMG38-2**	–	(92%+99.98%)	(98%+99.99%)
**pPvSC3**	(88%+99.98%)	–	(96%+99.75%)
**pM2-1**	(88%+99.99%)	(92%+99.75%)	–

Table S2. Pairwise comparison of plamid backbone sequences of pPvSC3-like plasmids using BLASTN.

(coverage+identity)	pNDM_NMG38-2	pPvSC3	pM2-1
**pNDM_NMG38-2**	–	(97%+99.98%)	(97%+99.99%)
**pPvSC3**	(96%+99.98%)	–	(99%+100%)
**pM2-1**	(96%+99.99%)	(99%+100%)	–

Table S3. Detailed information of *lnu(G)*-carrying plasmids or chromosomes (last accessed 18 Auguest, 2021）.

Accession no.	Plasmid/ chromosome	Host strain	Country	Year	Origin	Plasmid replicon
LR962611	chromosome	*Enterococcus faecalis*	Netherlands	2020	-	-
LR962628	chromosome	*Enterococcus faecalis*	Netherlands	2020	-	-
LR962531	chromosome	*Enterococcus faecalis*	Netherlands	2020	-	-
LR962454	chromosome	*Enterococcus faecalis*	Netherlands	2020	-	-
LR962846	chromosome	*Enterococcus faecalis*	Netherlands	2020	-	-
OD940420	chromosome	*Enterococcus faecalis*	United Kingdom	2021	Human	-
KX470419	chromosome	*Enterococcus faecalis*	China	2016	Animal	-
CP042216	chromosome	*Enterococcus faecalis*	Brazil	2013	Animal	-
CP043724	chromosome	*Enterococcus*	Brazil	2013	Animal	

		faecalis				
CP073085	chromosome	*Enterococcus faecalis*	Brazil	2013	Animal	
CP023074	chromosome	*Enterococcus thailandicus*	Canada	2017	Environment	-
CP075898	plasmid	*Exiguobacterium acetylicum*	China	2021	Fruit	NT
CP049753	chromosome	*Proteus mirabilis*	Brazil	2015	Human	-
CP054158	chromosome	*Providencia rettgeri*	Canada	2009	Food	-
MK840873	chromosome	*Enterococcus hirae*	China	2019	Animal	-
CP017962	chromosome	*Virgibacillus halodenitrificans*	China	2014	Environment	
CP064826	chromosome	*Morganella morganii*	China	2015	Human	
MT813046	plasmid	*Providencia stuartii*	China	2018-2019	Animal	NT
MG516911	plasmid	*Proteus mirabilis*	Australia	2017	Animal	NT
MH491967	plasmid	*Proteus mirabilis*	China	2018	-	NT
CP042907	chromosome	*Proteus mirabilis*	France	2016	Human	-
CP047640	plasmid	*Proteus sp.*	China	2020	Animal	NT
MW298655	plasmid	*Escherichia coli*	Canada	2020	Animal	NT
CP072987	plasmid	*Escherichia coli*	Canada	2020	Animal	NT
MW298657	plasmid	*Escherichia coli*	Canada	2020	Animal	NT
CP037911	plasmid	*Escherichia coli*	China	2017	Animal	IncX4, IncFIA(HI1), IncHI1A, IncHI1B(R27)
MN101856	plasmid	*Escherichia coli*	China	2019	Food	IncFIA(HI1), IncHI1A, IncHI1B(R27)
MN101858	plasmid	*Escherichia coli*	China	2019	Food	IncFII(pHN7A8), IncFIA(HI1), IncHI1A, IncHI1B(R27)
MW940615	plasmid	*Klebsiella sp.*	China	2019	Food	IncFIA(HI1), IncHI1A, IncHI1B(R27)
MT219825	plasmid	*Escherichia coli*	China	2020	Environment	IncX1,

						IncFIA(HI1), IncHI1A, IncHI1B(R27)
MW940627	plasmid	*Citrobacter sp.*	China	2019	Food	IncFIA(HI1), IncHI1A, IncHI1B(R27)
LT795503	plasmid	*Escherichia coli*	United Kingdom	2017	Animal	IncFIA(HI1), IncHI1A, IncHI1B(R27)
MK605407	plasmid	*Escherichia coli*	Czechia	2019	Animal	NT
CP046004	plasmid	*Escherichia coli*	China	2019	Animal	IncFIA(HI1), IncHI1A, IncHI1B(R27)
CP046007	plasmid	*Escherichia coli*	China	2019	Animal	IncFIA(HI1), IncHI1A, IncHI1B(R27)
CP059284	plasmid	*Escherichia coli*	China	2020	Food	IncFIA(HI1), IncHI1A, IncHI1B(R27)
CP058949	plasmid	*Escherichia coli*	China	2020	Food	IncFIA(HI1), IncHI1A, IncHI1B(R27)
CP059044	plasmid	*Escherichia coli*	China	2020	Food	IncFIA(HI1), IncHI1A, IncHI1B(R27)
CP046717	plasmid	*Escherichia coli*	China	2019	Animal	IncFIA(HI1), IncHI1A, IncHI1B(R27)
CP049354	plasmid	*Escherichia coli*	China	2020	Animal	IncFIA(HI1), IncHI1A, IncHI1B(R27)
CP041449	plasmid	*Escherichia coli*	China	2019	Food	IncFIA(HI1), IncHI1A, IncHI1B(R27)
MT219824	plasmid	*Escherichia coli*	China	2020	Animal	IncFIA(HI1), IncHI1A, IncHI1B(R27)
MW940625	plasmid	*Escherichia coli*	China	2019	Food	IncFIB(K), IncFIA(HI1), IncHI1A, IncHI1B(R27)
CP046026	plasmid	*Escherichia coli*	Canada	2019	Animal	NT
MK140641	plasmid	*Enterococcus faecalis*	China	2018	Animal	rep32

MT723965	plasmid	*Enterococcus faecalis*	Italy	2019	Animal	NT
AJ293027	plasmid	*uncultured eubacterium*	Germany	2000	Animal	NT
MW390518	plasmid	*Escherichia coli*	The Netherlands	2020	Animal	IncX4
MW390525	plasmid	*Escherichia coli*	The Netherlands	2020	Animal	IncX4
CP060813	plasmid	*Acinetobacter variabilis*	China	2020	Animal	NT
KY613742	plasmid	*Listeria monocytogenes*	Canada	2017	Food	rep25, repUS43
CP049889	chromosome	*Jeotgalibaca porci*	Spain	2017	Animal	-
KX774387	plasmid	*Providencia rettgeri*	Brazil	2016	Human	NT

NT: not typable.

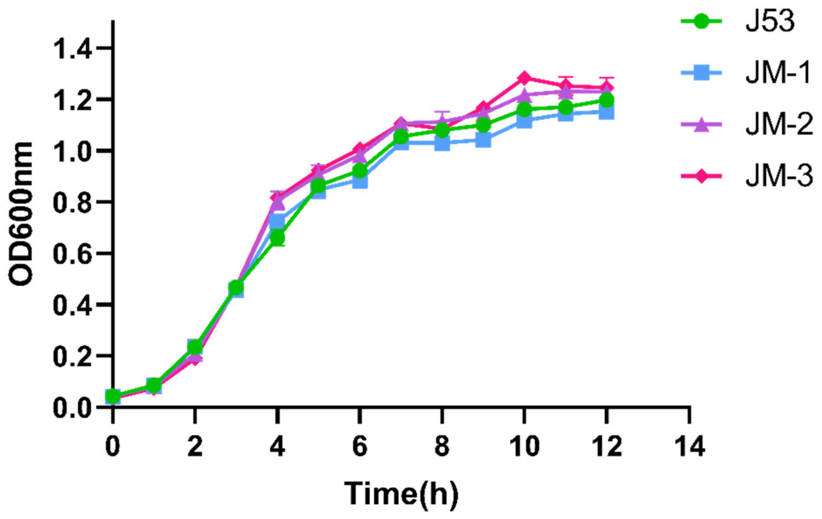

Figure S1. Growth curves for the transconjugants (JM-1, JM-2, and JM-3) and the recipient strain J53. Data were expressed as means ± standard deviations and error bars show SDs.
